# *Fusobacterium nucleatum* and *Treponema denticola* are robust biomarkers for gingivitis and periodontitis in small dogs

**DOI:** 10.3389/fvets.2024.1515521

**Published:** 2025-01-06

**Authors:** Daehyun Kwon, Kisuk Bae, Kwangsik Jang, Hyun Min Jo, Seong Soo Kang, Jonghoe Byun, Se Eun Kim

**Affiliations:** ^1^MAY Veterinary Dental Hospital, Seoul, Republic of Korea; ^2^Department of Veterinary Surgery, College of Veterinary Medicine and BK21 Plus Project Team, Chonnam National University, Gwangju, Republic of Korea; ^3^Department of Molecular Biology/Biological Sciences, School of Biomedical Sciences, Dankook University, Cheonan, Republic of Korea; ^4^Biomaterial R&BD Center, Chonnam National University, Gwangju, Republic of Korea

**Keywords:** periodontopathic bacteria, *Treponema denticola*, *Fusobacterium nucleatum*, biomarker, quantitative real-time PCR, canine gingivitis, canine periodontitis

## Abstract

**Introduction:**

Periodontal disease is one of the most common oral diseases in dogs and humans. It starts with gingivitis, a reversible condition, and progresses to an irreversible condition, periodontitis. Unlike humans, the etiology of periodontal disease in dogs has not been widely studied. Many studies suggest that bacteria strongly implicated in human periodontal disease might also play a role in canine periodontal disease. In contrast to studies examining only the prevalence of bacteria, a recent study analyzed 336 gingival crevicular fluid (GCF) samples in dogs to evaluate the prevalence of 11 putative periodontopathic bacteria and the correlation and association of bacterial numbers individually and in combination with periodontal disease stages. Results showed that *Treponema denticola* (Td) was a strong prognostic biomarker for periodontitis in dogs. However, a limitation of this study was that samples were grouped according to the periodontal status of the target tooth only, without assessment of the overall oral health. Furthermore, the findings of this study revealed a need for validation in a larger sample size.

**Materials and methods:**

This study ensured that the overall oral health assessment of dogs under 20 kg matched with sampled groups, thus eliminating the influence of environmental factors on the results. Furthermore, 1,054 GCF samples were analyzed using quantitative real-time polymerase chain reaction (qPCR) for 12 bacteria, including the same 11 putative periodontopathic bacteria [*Aggregatibacter actinomycetemcomitans* (Aa), *Porphyromonas gingivalis* (Pg), *Tannerella forsythia* (Tf), Td, *Fusobacterium nucleatum* (Fn), *Prevotella nigrescens* (Pn), *Prevotella intermedia* (Pi), *Parvimonas micra* (Pm), *Eubacterium nodatum* (En), *Campylobacter rectus* (Cr), and *Eikenella corrodens* (Ec)] and *Porphyromonas gulae* (*P. gulae*), suspected to be a major causative agent of periodontitis in dogs in some statistical evaluatioins.

**Results:**

Interestingly, the present study found that Fn was strongly associated with gingivitis and reconfirmed a strong association between Td and periodontitis (irreversible periodontal disease). However, Aa showed no relevance, and *P. gulae* was not significantly associated with periodontal disease in dogs in this study.

**Conclusion:**

These findings suggest that Fn and Td would be robust biomarkers for the severity of periodontal disease in small dogs.

## 1 Introduction

Periodontal disease is one of the most common oral diseases found in dogs worldwide (1–4). Clinically, it starts with gingivitis, a reversible inflammatory condition characterized by swelling and redness of the gums, and gradually progresses to periodontitis, an irreversible disease state characterized by the destruction of tooth-supporting structures, such as periodontal ligament and alveolar bone (5, 6). Periodontal disease is caused by a complex interaction between gingival crevicular fluid (GCF) bacteria, host, and environmental factors (4).

While the relationship between periodontal disease and subgingival bacteria in humans has been well-studied for decades, it has not been widely studied in dogs. Many researchers have suspected human periodontitis-related bacteria are putative periodontitis-related pathogens in dogs (7, 8). However, most studies have used small sample sizes, providing less generalizability of results (9, 10). Furthermore, most studies have analyzed only the presence of putative bacteria, lacking information on changes in bacterial counts among reversible (healthy and gingivitis) and irreversible (periodontitis) periodontal conditions and different stages of periodontitis in dogs.

Recently, Kwon et al. performed quantitative real-time polymerase chain reaction (qPCR) for 11 putative periodontopathic bacterial species in dogs using 336 GCF samples. Correlations and associations between the number of bacterial species and various periodontal conditions were analyzed based on the qPCR results. They showed that *Aggregatibacter actinomycetemcomitans* (Aa) and *Porphyromonas gingivalis* (Pg), the representative periodontopathic bacteria in humans, were not associated with periodontitis in dogs. *Treponema denticola* (Td) was strongly correlated and associated with periodontitis in dogs, serving as a prognostic biomarker (11).

However, studies using larger sample sizes are necessary for accurate results. Moreover, the effect of different sampling spots on teeth and the oral environment needs consideration. The study examined 336 samples grouped according to the periodontal status of a single tooth used for sample collection (6 test sites per tooth were sampled and pooled). However, it did not assess the influence of the overall oral environment. Hence, this study selected 1,286 teeth from 643 dogs and grouped them to ensure that oral environmental factors did not influence the results.

Furthermore, *Porphyromonas gulae* (*P. gulae*), a possible causative agent of periodontal disease in dogs (based on its prevalence in several studies) (12–14), was analyzed with 11 bacterial species evaluated in the previous study (11). The 12 bacterial species examined were Aa, Pg, *Tannerella forsythia* (Tf), Td, *Fusobacterium nucleatum* (Fn), *Prevotella nigrescens* (Pn), *Prevotella intermedia* (Pi), *Parvimonas micra* (Pm), *Eubacterium nodatum* (En), *Campylobacter rectus* (Cr), *Eikenella corrodens* (Ec), and *P. gulae*. This study used qPCR and explored whether bacterial prevalence of the 12 putative periodontopathic bacteria (increase and/or decrease individually and in combination) has a significant association with severity of periodontal disease in small dogs.

## 2 Materials and methods

### 2.1 Grouping of 643 dogs

The study used client-owned dogs that underwent various dental treatments, such as periodontal therapy, endodontic treatment, simple scaling, and general oral examination, at MAY Veterinary Dental Hospital. The owners were informed of the purpose of the study and signed an informed consent to obtain samples with paper points from their dogs during the procedure. All procedures were performed under general inhalation anesthesia, and every effort was made to minimize pain through non-invasive methods. The dogs were evaluated through serum biochemistry tests, complete blood count (CBC), chest radiography, auscultation, and blood pressure measurements before the scheduled dental procedure, and had not received antibiotics in the previous 3 months.

Willas et al. hypothesized that the prevalence of periodontal disease would vary across breed size categories, breeds, and body weight and found that most breeds diagnosed with periodontal disease were in the extra-small (<6.5 kg), small (6.5–9 kg), and medium-small (9–15 kg) size categories (15). Based on these findings, dogs used in the study were limited to those weighing less than 20 kg, which is common in Korea, even if this weight restriction prevents the results from being representative of the entire dog population.

**Figure 1:**
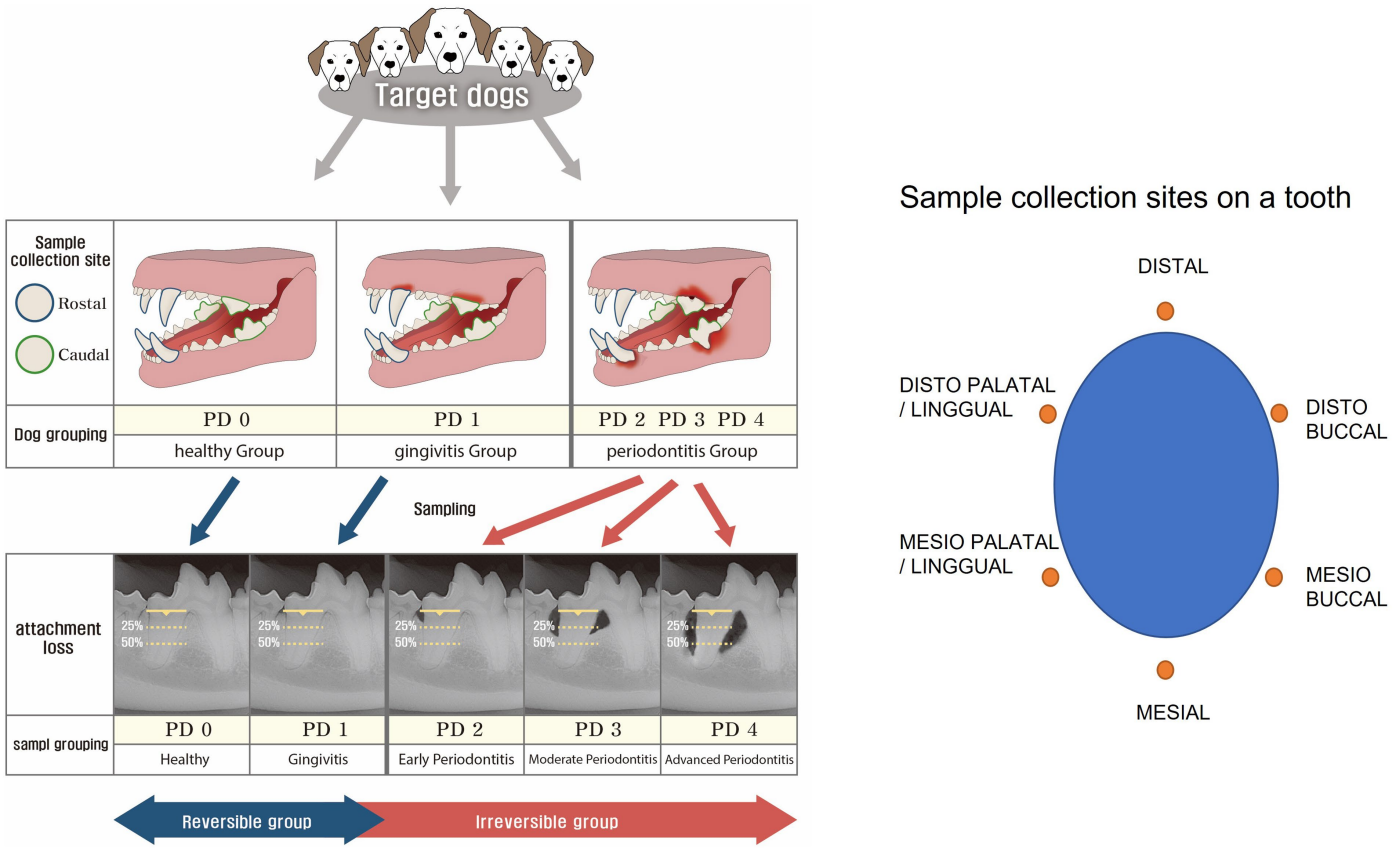
Grouping criteria and sample collection sites. The dogs were initially classified into three groups (healthy, gingivitis, and periodontitis). Then, two teeth on each dog were selected for further classification into five periodontal disease (PD) groups based on clinical conditions, such as healthy (PD0), gingivitis (PD1), early periodontitis (PD2), moderate periodontitis (PD3), and advanced periodontitis (PD4). While the reversible group comprised PD0 and PD1, the irreversible group comprised PD2, PD3, and PD4. Then, samples were collected at six points around a tooth.

Strict categorization was applied to minimize the impact of confounding factors that could affect the results. The 643 dogs under 20 kg were assessed for overall oral condition and categorized into three groups based on the probing of all teeth in the oral cavity and full-mouth intraoral radiographic evaluation as follows: Healthy group: healthy overall periodontal condition. Gingivitis group: the worst periodontal condition was gingivitis only. Periodontitis group: periodontitis involving one or more teeth (Figure 1).

### 2.2 Sample collection

The study used 1,286 subgingival GCF samples from 643 dogs over 1 year between January 2020 and December 2023. Among the samples analyzed in this study, only 36 samples from 18 dogs collected over 2 months, from January to February 2020, overlap with those used in the 2022 study by Kwon et al. (11). The general anesthesia protocol was as follows: premedication with subcutaneous glycopyrrolate (0.01 mg/kg Mobinul; Myungmoon Pharm., Seoul, Korea); intravenous butorphanol (0.1 mg/kg Bu; Myungmoon, Gyeonggi, Korea) and midazolam (0.2 mg/kg Midacom; Myungmoon Pharm.). Propofol (4 mg/kg Probio; Myungmoon Pharm.) was administered intravenously for induction, and anesthesia was maintained with sevoflurane at 2.3% and O_2_ at 2 L/min followed by the placement of a cuffed endotracheal tube. Lactated Ringer’s solution was administered intravenously at 10 mL/kg/h throughout the procedure. A conductive-fabric patient warming system was placed under the dogs, and they were monitored using combination monitoring equipment.

The sampling sites were divided into rostral and caudal teeth of the oral cavity. Rostral target teeth were mandibular and maxillary canines, which are functionally important rostral teeth. Maxillary fourth molar and mandibular first molar teeth (primary masticatory teeth) were categorized as caudal. Samples were taken from two teeth found most periodontally compromised in each dog (Figure 1).

Before applying the oral cleansing agent (chlorhexidine), six sterile paper points (International Organization for Standardization #30) were gently inserted into the six subgingival pockets (distal, distobuccal, mesiobuccal, mesial, mesiopalatal/lingual, and distopalatal/lingual) around the target tooth for 30 s to obtain GCF samples. These six paper points were immediately transferred to a sterile transport tube, assigned a unique barcode number, and analyzed as one sample. The pooled samples were stored at 4°C until DNA extraction.

### 2.3 Grouping of target teeth by the 5 stages of periodontal disease

The American Veterinary Dental College describes five stages of periodontal disease as follows: healthy (PD0), gingivitis (PD1), early periodontitis (PD2), moderate periodontitis (PD3), and advanced periodontitis (PD4), based on the severity of attachment loss (16). Intraoral dental radiographs were obtained under general anesthesia and evaluated by the same veterinarian (Daehyun Kwon) (under consistent conditions) using a standard approach.

A total of 1,286 target teeth from 643 dogs were categorized into PD0, PD1, PD2, PD3, and PD4 based on their periodontal conditions. Among them, teeth from dogs in the healthy group were categorized as PD0. In the gingivitis group, one or two teeth involved in gingivitis were classified as PD1. Similarly, among teeth in the periodontitis group, only those exhibiting periodontitis according to radiological evaluation were categorized into PD2, PD3, and PD4 groups; teeth without periodontitis were excluded. PD0 and PD1 constituted the reversible group, while PD2, PD3, and PD4 comprised the irreversible group. The reversible group was strictly defined by the absence of periodontitis in the oral cavity, and the PD0 group was strictly defined by the absence of any periodontal disease in the oral cavity (Figure 1). The reversible group was differentiated from the irreversible group by the absence of intraoral periodontitis, and the PD0 group was strictly separated from the other sample groups (PD1 to PD4) by the absence of intraoral periodontal disease. Finally, 1,054 GCF samples that met the strict grouping criteria described above were analyzed and evaluated.

### 2.4 DNA extraction and storage

DNA extraction from the GCF samples was performed using an Exgene™ Cell SV kit (GeneAll Biotechnologies, Seoul, Korea), following the manufacturer’s instructions. The paper point was treated with 180 μL of lysozyme at 30 mg/mL and incubated at 37°C for 30 min. Proteinase K solution (20 μL of 20 mg/mL) and 200 μL of buffer BL were added to each sample, followed by incubation at 56°C for 30 min and 95°C for 15 min. Subsequently, 200 μL of absolute ethanol was added, and the mixture was transferred into a column and centrifuged at 14,000 rpm for 1 min. After washing the column with 600 μL of buffer BW, 700 μL of buffer TW was added. Next, 100 μL of buffer AE was used to elute DNA. DNA was quantified with a NanoDrop spectrophotometer (Thermo Fisher Scientific, Inc., Waltham, MA, United States). DNA samples were stored at −20°C until use.

### 2.5 Quantitative real-time polymerase chain reaction assay

The targeted oral bacteria and primer/probe set sequences used for quantitative real-time PCR are listed in Table 1. PCR amplification was performed in a reaction volume of 20 μL (Bioneer, Inc., Daejeon, Korea). PCR cycling was conducted using a CFX96™ real-time system (Bio-Rad Laboratories Inc., Hercules, CA, United States). Cycling conditions consisted of an initial denaturation step at 95°C for 5 min, followed by 40 cycles of denaturation at 95°C for 30 s, primer annealing at 60°C for 40 s, and primer extension at 72°C for 30 s. After completing the cycling steps, a final extension step at 72°C for 5 min was performed. The normalized expression value for each species was calculated as the ratio of the relative copy number of the reference species. The qPCR performed with the primer-probe sets used in this study is shown in Table 1.

**Table 1:** Target oral bacteria and primer/probe sequences used for quantitative real-time PCR.

Pathogens and primer/probe	Primer/probe set sequence (5′ to 3′)	Length (Base)	Amplicon size (bp)	Reference
Aa AaLtF14 AaLtR11 AaLtP13	CGGTGGAGAAGGAAATGATATTTATG ATTGCCGTTACGCTCAAATG FAM-CCACACTATTACGGAACATAGCGGTG-BHQ-1	26 20 28	139	Kwon et al. (11)
Pg PghaF14 PghaR13 PghaP11B	GCAGGGTCAGAAAGTAACGCTC CGATCCGTTTTACTTCACGG HEX-CCGAGCGCAAAGAAGGCAGAA-BHQ-1	22 20 21	80	Kwon et al. (11)
Tf TfKpF13 TfKpR12 TfKpP11	CCGGCGGTTTCCTGTAGTAGA ACTTCGTCCGTTGCAGGGTT TEXAS RED-CTCCCTTCACCCTCTCGCCG-BHQ-2	21 20 20	68	Kwon et al. (11)
Td TdopF13 TdopR13 TdopP01	CATCTCTTGATGCAGCCGAAG GTCAGGGCTTACAACATAGTCGTC Cy5-TGGCGGAAGGAAAACAAGCC-BHQ-2	21 24 20	98	Kwon et al. (11)
Fn. FnChF15 FnChR13 FnChP12	GACATCTTAGGAATGAGACAGAGATG CAGCCATGCACCACCTGTCT TEXAS RED-CAGTGTCCCTTCGGGGAAACCT-BHQ-2	26 20 22	73	Kwon et al. (11)
Pn PngyF12 PngyR13 PngyP11	GCAAGAACGTGATGACGGGA ATTTCGCAGTCTTTGGGATCT TT Cy5-TTGCCAGGAAAACTTGCCGA-BHQ-2	20 23 20	79	Kwon et al. (11)
Pi PipiF12 PipiR13 Pi194P13H	CCACCAACGACAACCTTCCA TCTACTGCTTCGAGCGCAC HEX-CAAGACAATCTCCGACGGAACGTT-BHQ-1	20 19 24	103	Kwon et al. (11)
En EnglF01 EnglR01 EnglP01	ATCCACAACAAAAGCGGCCT AGGAATGTCCGGAGCAGGAA HEX-CAAACCAATCTGCAGCATGGG-BHQ-1	20 20 21	157	Kwon et al. (11)
Pm PmF-30 PmR-30 Pm16S30	AAACGACGATTAATACCACATGAGAC ACTGCTGCCTCCCGTAGGA TEXAS RED-TCAAAGATTTATCGGTGTAAGAAGGGCTCGC-BHQ-2	26 19 31	201	Nonnenmacher et al. (42)
Cr CrgrF14 CrgrR12 CrgrP01	GCGAAGTAGTGAGCGAAGAG GCCTGCGCCATTTACGATA FAM-CAAGCGTGATCATCGACAAGGATAACA-BHQ-1	20 19 27	119	Kwon et al. (11)
Ec EcISRF-21 EcISRR-21 EcISRP21	AGGCGACGAAGGACGTGTAA ATCACCGGATCAAAGCTCTATTG Cy5-CGTGTAAGCCTGCGAAAAGCATCG-BHQ-2	20 23 24	69	Kwon et al. (11)
*P. gulae* PgulF1 PgulR1	CAGGGAGCCAATACGACGAT CGCCTCATATGCCACCTTGA	20 20	150	This study

Aa, *Aggregatibacter actinomycetemcomitans*, Pg: *Porphyromonas gingivalis*, Tf: *Tannerella forsythia*, Td: *Treponema denticola*, Fn: *Fusobacterium nucleatum*, Pn: *Prevotella nigrescens*, Pi: *Prevotella intermedia*, En: *Eubacterium nodatum*, Pm: *Parvimonas micra*, Cr: *Campylobacter rectus*, Ec: *Eikenella corrodens*, *P. gulae*: *Porphyromonas gulae*, F: Forward primer, R: Reverse primer, P: Probe.

### 2.6 Data management and statistical analysis

Data management and statistical analysis were conducted using MedCalc® Statistical Software, version 22.009.^1^ The independence of sample characteristics was analyzed using independent t-tests. The correlation of the number of bacteria, age, weight, bleeding on probing (BOP), and probing pocket depth (PPD) with the severity of the periodontal condition was evaluated using Kendall’s tau correlation coefficient. When measuring PPD, gingival enlargement was excluded, but gingival recession was included. However, measurements were recorded as ‘2/5’ to distinguish a 2 mm pocket from a 3 mm gingival recession to equal the attachment loss as 5 mm. The correlation between the number of bacteria and age, weight, BOP, and PPD was assessed using Pearson’s r correlation coefficient. Associations of each bacterium or combination of bacteria between reversible and irreversible groups were analyzed using logistic regression. The significance level was set at *p* < 0.05.

## 3 Results

### 3.1 Characteristics of dogs with gingival crevicular fluid sampling

Table 2 describes the characteristics of dogs. Compared to the irreversible group, the mean age was lower (reversible vs. irreversible group: 5.44 ± 2.80 vs. 8.39 ± 3.70), and conversely, the mean weight was higher in the reversible group (reversible vs. irreversible group: 8.78 ± 5.32 vs. 6.17 ± 3.73). There was no significant difference in sex between the two groups (51.0% males in the reversible group and 48.6% in the irreversible group).

**Table 2:** Demographic profiles of dogs that provided the gingival crevicular fluid (GCF) samples.

	Reversible group	Irreversible group	P
Age (years)	5.44 ± 2.80	8.39 ± 3.70	<0.0001
Male (%)	51.0	48.6	0.4527
Weight (kg)	8.78 ± 5.32	6.17 ± 3.73	<0.0001

Data of age and weight were analyzed using independent t-test and given as mean ± standard deviation.

### 3.2 Prevalence of putative periodontal disease-related bacteria in dogs

**Figure 2:**
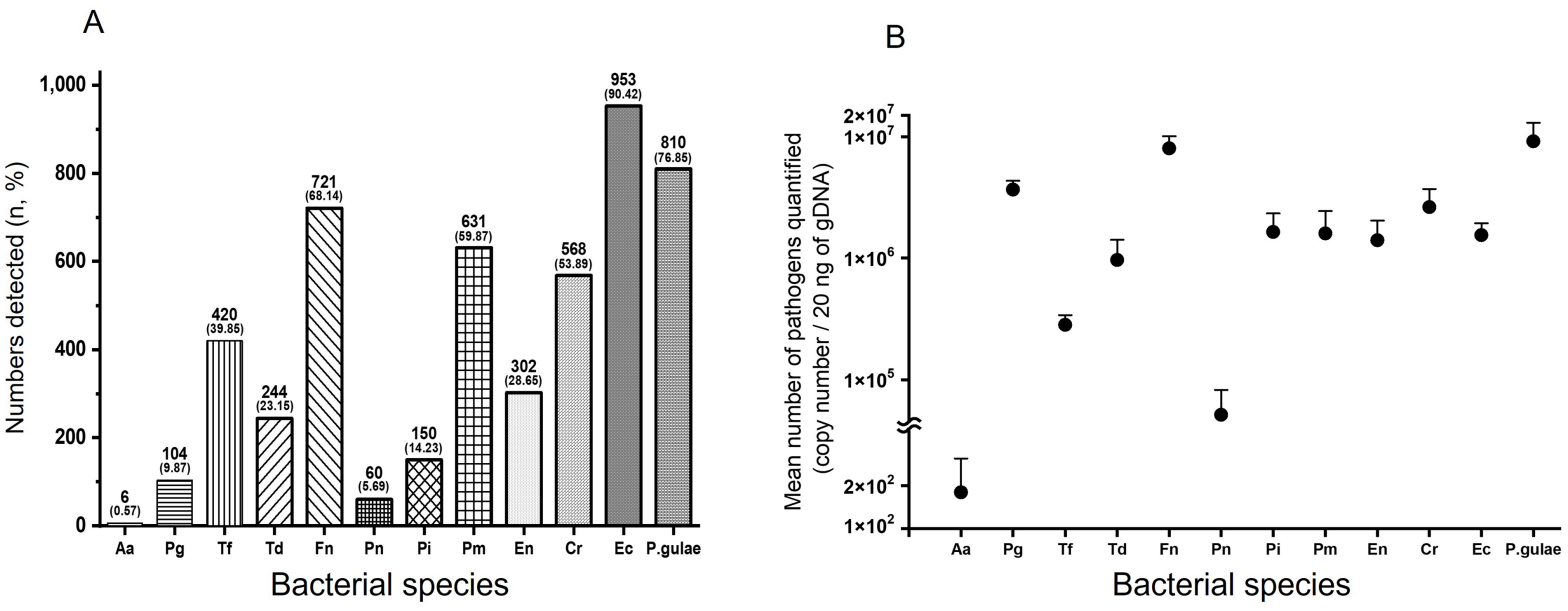
Relative prevalence and mean number of bacteria. Overall prevalence of bacteria **(A)**. Bars represent the number of detected bacterial species and their percentages (%). Overall mean number of bacteria **(B)**. Bars show standard deviation. Log = logarithm. Aa: *Aggregatibacter actinomycetemcomitans*, Pg: *Porphyromonas gingivalis*, Tf: *Tannerella forsythia*, Td: *Treponema denticola*, Fn: *Fusobacterium nucleatum*, Pn: *Prevotella nigrescens*, Pi: *Prevotella intermedia*, Pm: *Parvimonas micra*, En: *Eubacterium nodatum*, Cr: *Campylobacter rectus*, Ec: *Eikenella corrodens*, *P.gulae*: *Porphyromonas gulae*.

Figure 2A shows the overall prevalence of putative periodontal disease-related bacteria in dogs. The overall prevalence of Aa was very low at 0.57%, and the prevalence of Ec was the highest at 90.42%; Fn, Pm, Cr, Ec, and *P. gulae* showed an overall prevalence rate of over 50%.

### 3.3 Comparison of bacterial prevalence between reversible and irreversible groups

**Figure 3:**
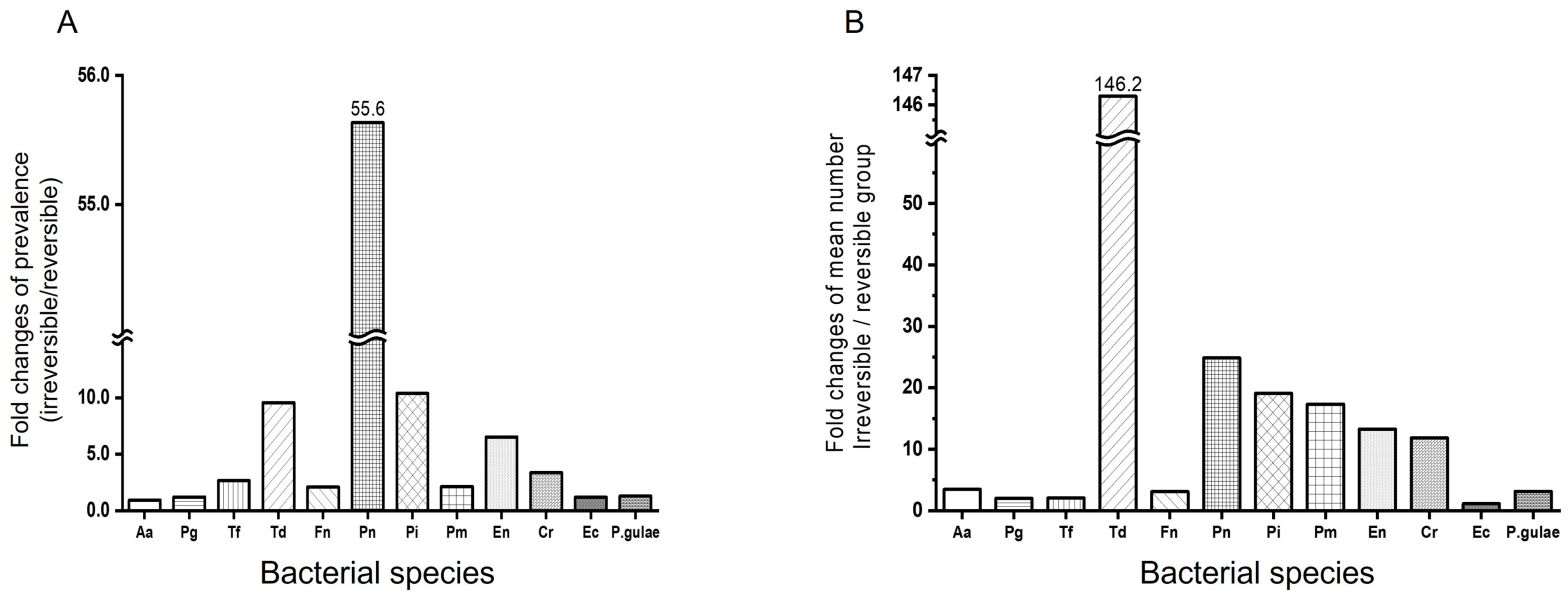
Comparison of reversible and irreversible groups for bacterial prevalence and mean number. Comparison of reversible and irreversible groups for bacterial prevalence. Prevalence **(A)** and mean number **(B)** of pathogenic bacteria were compared between reversible and irreversible groups. The five stages of periodontal disease are as follows: healthy (PD0), gingivitis (PD1), early periodontitis; less than 25% of attachment loss (PD2), moderate periodontitis; 25–50% of attachment loss (PD3), and advanced periodontitis; more than 50% attachment loss (PD4). Reversible group: PD0, PD1; Irreversible group: PD2, PD3, and PD4. Aa: *Aggregatibacter actinomycetemcomitans*, Pg: *Porphyromonas gingivalis*, Tf: *Tannerella forsythia*, Td: *Treponema denticola*, Fn: *Fusobacterium nucleatum*, Pn: *Prevotella nigrescens*, Pi: *Prevotella intermedia*, Pm: *Parvimonas micra*, En: *Eubacterium nodatum*, Cr: *Campylobacter rectus*, Ec: *Eikenella corrodens*, *P.gulae*: *Porphyromonas gulae*.

Figure 3A compares bacterial prevalence among different groups. First, in the difference of prevalence between the reversible and irreversible groups, Pn shows the most overwhelming difference (55.68-fold) in the two groups, followed by Pi (10.38-fold), Td (9.56-fold), and En (6.52-fold). Aa, Pg, Ec, and *P. gulae* had a small difference of less than 2-fold, with Aa having the smallest difference in prevalence between the reversible and irreversible groups at 0.93-fold.

### 3.4 Mean number of putative periodontal disease-related bacteria in dogs

Except for Aa and Pn, the overall mean number of each of the 10 bacteria was over 100,000. Among them, *P. gulae* was the most abundant, with 9,144,012 ± 3,883,395, followed by Fn with 8,017,759 ± 2,058,234. Aa had the lowest overall mean number of bacteria (179 ± 131), significantly lower than the other three bacteria (Figure 2B).

### 3.5 Comparison of mean number of bacteria between reversible and irreversible groups

Td alone showed a significant variation when comparing each mean bacterial number between the reversible and irreversible groups. Td showed a 146.29-fold (*p*-value <0.001) increase in bacterial number in the irreversible group compared to the reversible group. Pn, Pi, Pm, En, and Cr increased by 10 times or more in the irreversible group compared to the reversible group, with the increase of bacterial number being 24.86 times, 19.09 times, 17.35 times, 13.29 times, and 11.83 times, respectively (*p*-value = 0.2108, <0.0001, <0.0001, <0.0001, and < 0.0001, respectively). Aa, Pg, Tf, Fn, Ec, and *P. gulae* showed increased counts in the irreversible group compared to the reversible group, but the increase was less than 4-fold (3.47 times, 1.99 times, 2.04 times, 3.10 times, 1.13 times, and 3.13 times, respectively; *p*-value = 0.3708, 0.0273, 0.0286, <0.0001, 0.2196, and 0.0231, respectively; Figure 3B).

### 3.6 Correlation of between severity of periodontal disease and age, weight, BOP, mean PPD, and number of 12 bacteria

Older age (*p*- value <0.0001/OR = 0.319) and lower body weight (*p*- value <0.0001/OR = −0.196) were significantly correlated with periodontal disease severity. Increased BOP (*p*- value <0.0001/OR = 0.444) and mean PPD (*p*- value <0.0001/OR = 0.786) were also significantly associated with periodontal disease severity in dogs. However, only Aa (*p*- value = 0.8586), among the 12 bacteria, did not correlate with the severity of periodontal disease in dogs (Table 3). Increased BOP and PPD showed a significant correlation with the severity of periodontal disease in dogs. Therefore, BOP and PPD were excluded from further analyses of the association with periodontal disease across a combination of age, weight, and number of bacteria.

**Table 3:** Correlation between severity of periodontal disease and age, weight, BOP, mean PPD, and number of 12 bacteria present.

Target factor	Odds ratio (95% CI)	p-value
Age	0.319 (0.281–0.356)	<0.0001
Weight	−0.196 [−0.240-(−0.153)]	<0.0001
BOP (%)	0.444 (0.406–0.482)	<0.0001
Mean PPD (mm)	0.786 (0.766–0.802)	<0.0001
Aa	−0.00365 (−0.0431–0.0437)	0.8586
Pg	0.0697 (0.0249–0.117)	0.0007
Tf	0.309 (0.264–0.351)	<0.0001
Td	0.327 (0.289–0.363)	<0.0001
Fn	0.439 (0.400–0.481)	<0.0001
Pn	0.219 (0.175–0.259)	<0.0001
Pi	0.273 (0.229–0.313)	<0.0001
Pm	0.414 (0.370–0.456)	<0.0001
En	0.372 (0.327–0.407)	<0.0001
Cr	0.465 (0.428–0.498)	<0.0001
Ec	0.208 (0.166–0.260)	<0.0001
*P. gulae*	0.281 (0.238–0.320)	<0.0001

BOP: Bleeding on probing, PPD: Probing pocket depth, Aa: *Aggregatibacter actinomycetemcomitans*, Pg: *Porphyromonas gingivalis*, Tf: *Tannerella forsythia*, Td: *Treponema denticola*, Fn: *Fusobacterium nucleatum*, Pn: *Prevotella nigrescens*, Pi: *Prevotella intermedia*, En: *Eubacterium nodatum*, Pm: *Parvimonas micra*, Cr: *Campylobacter rectus*, Ec: *Eikenella corrodens*, *P. gulae*: *Porphyromonas gulae*.

### 3.7 Correlation of Aa in all possible between-group comparisons

Statistical analyses performed in all possible between-group comparisons confirmed that Aa did not correlate with the and severity of periodontal disease in dogs (the data is not shown.). Therefore, Aa was excluded from subsequent analyses of the association between periodontal disease in a combination of age, weight, and number of bacteria.

### 3.8 Association of the combined coexistence of age, weight, and the number of bacteria between reversible and irreversible groups

The association of age, weight, and the number of 11 bacteria (except for Aa) was analyzed between the reversible group and each different comparison group in the combination (Table 4). In the comparison between the reversible and irreversible groups, Pn (*p*-value = 0.0617), En (*p*-value = 0.1107), and *P. gulae* (*p*- value = 0.8232) showed no statistically significant associations. However, Td (*p*-value = 0.0002/OR = 1.1221) and Fn (*p*-value <0.0001/OR = 1.1065) showed a relatively robust association. In the comparison between the reversible and PD2 groups, Pn (*p*-value = 0.2284), En (*p*-value = 0.2913), Cr (*p*- value = 0.0549), and *P. gulae* (*p*-value = 0.7823) showed no association, while Td (*p*- value = 0.0002 / OR = 1.1335) showed a robust association.

**Table 4:** Association of the combined coexistence of age, weight, and the number of bacteria between reversible and irreversible groups.

	Target factor	Odds ratio (95% CI)	*p*- value
Reversible vs. Irreversible	Age	1.1372 (1.0711–1.2073)	<0.0001
	Weight	0.9317 (0.8942–0.9707)	0.0007
	Pg	0.9122 (0.8693–0.9572)	0.0002
	Tf	1.076 (1.0361–1.1175)	0.0001
	Td	1.1221 (1.0563–1.1921)	0.0002
	Fn	1.1065 (1.0737–1.1402)	<0.0001
	Pi	1.0945 (1.0273–1.1659)	0.0052
	Pm	1.0504 (1.0138–1.0883)	0.0066
	Cr	1.0541 (1.0199–1.0895)	0.0017
	Ec	1.0665 (1.0045–1.1323)	0.0351
Reversible vs. PD2	Age	1.0939 (1.0196–1.1735)	0.0124
	Weight	0.9498 (0.9074–0.9942)	0.0271
	Pg	0.9267 (0.8783–0.9778)	0.0054
	Tf	1.0605 (1.0177–1.1052)	0.0052
	Td	1.1335 (1.0620–1.2098)	0.0002
	Fn	1.0817 (1.0456–1.1189)	<0.0001
	Pi	1.0735 (1.0002–1.1522)	0.0493
	Pm	1.051 (1.0093–1.0945)	0.0161
	Ec	1.0715 (1.0036–1.1441)	0.0386
Reversible vs. PD3	Age	1.1717 (1.0684–1.2851)	0.0008
	Weight	0.9335 (0.8770–0.9937)	0.0309
	Pg	0.9 (0.8362–0.9687)	0.005
	Tf	1.1207 (1.0615–1.1833)	<0.0001
	Td	1.1201 (1.0419–1.2041)	0.0021
	Fn	1.1252 (1.0670–1.1865)	<0.0001
	Pi	1.1298 (1.0482–1.2178)	0.0014
	Cr	1.0868 (1.0374–1.1386)	0.0005
Reversible vs. PD4	Age	1.2298 (1.1219–1.3481)	<0.0001
	Weight	0.8446 (0.7734–0.9225)	0.0002
	Pg	0.8711 (0.8069–0.9405)	0.0004
	Tf	1.0954 (1.0322–1.1625)	0.0027
	Td	1.1519 (1.0649–1.2460)	0.0004
	Fn	1.1835 (1.1073–1.2649)	<0.0001
	Pn	1.2554 (1.0116–1.5580)	0.0389
	Pi	1.0954 (1.0154–1.1818)	0.0186
	Cr	1.0702 (1.0128–1.1310)	0.0159

The five stages of periodontal disease are as follows: healthy (PD0), gingivitis (PD1), early periodontitis; less than 25% of attachment loss (PD2), moderate periodontitis; 25–50% of attachment loss (PD3), and advanced periodontitis; more than 50% attachment loss (PD4). Reversible group: PD0, PD1, Irreversible group: PD2, PD3, and PD4, Pg: *Porphyromonas gingivalis*, Tf: *Tannerella forsythia*, Td: *Treponema denticola*, Fn: *Fusobacterium nucleatum*, Pn: *Prevotella nigrescens*, Pi: *Prevotella intermedia*, Cr: *Campylobacter rectus*.

Pn (*p*- value = 0.0979), Pm (*p*- value = 0.1607), En (*p*- value = 0.3203), Ec (*p*- value = 0.2818), and *P. gulae* (*p*- value = 0.5929) showed no association between reversible and PD3 groups, while Tf (*p*- value <0.0001/OR = 1.1207), Td (*p*- value = 0.0021 / OR = 1.1201), Fn (*p*- value <0.0001/OR = 1.1252), and Pi (*p*- value = 0.0014/OR = 1.1298) showed robust associations.

In the comparison between reversible and PD4 groups, Td (*p*-value = 0.0004/OR = 1.1519), Fn (*p*- value <0.0001/OR = 1.1835), and Pn (*p*- value = 0.0389/OR = 1.2554) showed substantial associations, while Pm (*p*- value = 0.2721), En (*p*- value = 0.1621), Ec (*p*- value = 0.9642), and *P. gulae* (*p*- value = 0.9929) showed no association. Td and Fn showed a robust association in all comparison conditions. However, *P. gulae* was not associated with any comparison condition. Also, while all other factors were associated with an increase, weight and Pg were associated with a decrease (both OR were less than 1.0 in all comparison conditions).

### 3.9 Association of the combined coexistence of age, weight, and the number of bacteria between each PD group

Table 5 showed that Fn was associated in all comparisons between each PD group, except between PD3 and 4 groups; Td (*p*- value = 0.004/OR = 1.127) had the strongest statistical association in the comparison between PD1 and PD2 groups. The comparison between PD3 and PD4 groups revealed no statistically significant bacteria.

**Table 5:** Statistical significance of the association between the combined coexistence of age, weight, and the number of bacteria across each periodontal disease (PD) group.

	Target factor	Odds ratio (95% CI)	p-value
PD0 vs. PD1	Weight	1.0616 (1.0126–1.1129)	0.0131
	Fn	1.0562 (1.0149–1.0991)	0.0072
	Ec	1.1112 (1.0361–1.1917)	0.0032
PD1 vs. PD2	Weight	0.9156 (0.8662–0.9678)	0.0018
	Pg	0.9234 (0.8687–0.9815)	0.0104
	Td	1.127 (1.0389–1.2225)	0.004
	Fn	1.0482 (1.0056–1.0925)	0.0261
PD2 vs. PD3	Fn	1.0601 (1.0155–1.1068)	0.0078
	Pi	1.0414 (1.0006–1.0838)	0.0466
	Cr	1.0465 (1.0085–1.0860)	0.0159
PD3 vs. PD4	Age	1.073 (1.0063–1.1441)	0.0313

The five stages of periodontal disease are as follows: healthy (PD0), gingivitis (PD1), early periodontitis; less than 25% of attachment loss (PD2), moderate periodontitis; 25–50% of attachment loss (PD3), and advanced periodontitis; more than 50% attachment loss (PD4). Fn: *Fusobacterium nucleatum*, Ec: *Eikenella corrodens*, Pg: *Porphyromonas gingivalis*, Td: *Treponema denticola*, Pi: *Prevotella intermedia*, Cr: *Campylobacter rectus*.

## 4 Discussion

Periodontal disease is one of the most common oral disorders, affecting up to 80% of adult dogs (10). All breeds of dogs are at risk for periodontal disease (13). Periodontitis, an irreversible periodontal disease, affects approximately 44–63.6% of dogs, with its prevalence and severity increasing with age and smaller body weight (2, 17). By 4 years, around 80% of dogs exhibit signs of periodontitis, escalating to 96% by the age of 12–14 (18). Furthermore, recent research has confirmed that extra-small breeds are five times more prone to periodontal disease compared to giant breeds in dogs, with poodles (miniature and toy) showing the highest statistically significant incidence of periodontal disease (15). Periodontitis is categorized into three grades based on the degree of attachment loss: early, moderate, and advanced periodontitis (16). Understanding the causes of periodontitis is crucial for predicting and preventing irreversible conditions.

In humans, extensive research on periodontitis-causing bacteria has focused on the dysbiosis of subgingival biofilms (8, 19–22). Studies have reported that bacterial species colonizing the periodontal pocket play different roles in disease pathogenesis (19, 23–25). Among the hundreds of bacterial species, only a few are involved in disease development and progression (24). Pg, a gram-negative anaerobic rod, disrupts the complement system and triggers complement-dependent inflammation, compromising host response. This disruption leads to dysbiosis, altering the composition of commensal microbiota and creating an environment conducive to the proliferation of dysbiotic bacteria, thereby accelerating periodontal disease progression. Pg has been identified as a keystone pathogen in human periodontitis (19). However, such specific hypotheses have not been proposed, nor have specific bacteria been identified to predict the severity of periodontal disease in dogs.

Recent studies have revealed that the canine oral microbiome is significantly different from the human oral microbiome, with bacteria absent or rare in human subgingival plaque detectable at notably high levels in the subgingival plaque of dogs (26–28). Dewhirst et al. identified 353 canine oral bacterial taxa from 51 dogs and analyzed their characteristics. They discovered that only 16.4% of the oral bacterial taxa were shared between dogs and humans, while 83.6% of the taxa found in dogs were novel (28). However, it would also be meaningful to investigate whether there are specific bacteria shared between humans and dogs that have a clear correlation and association with periodontal disease in dogs. Therefore, many studies have suggested that bacteria implicated in human periodontitis may also play a role in dogs. Despite the differences in the microbiome and environmental factors between dogs and humans, most human periodontitis-associated bacterial species are also suspected to be putative pathogens in dogs (7–11, 13, 29–31). Most studies were conducted using small sample sizes, although Kwon et al. analyzed a larger sample size to evaluate the prevalence, abundance, and association of 11 putative periodontopathic bacteria in dogs (11). However, their study needs validation in larger sample sizes. Moreover, because the periodontal status of only the target teeth was assessed and grouped, one cannot completely rule out the possibility that the overall oral environment affected the outcome. Therefore, the present study used a larger sample size (1,054 teeth) than previous studies (3.14-fold larger, Kwon’s paper (11)) and grouped the samples following strict criteria. Moreover, this study also analyzed *P. gulae* to confirm its correlation or association with periodontitis in companion animals, as shown in several studies (7, 12, 14, 32). The 12 bacterial species highlighted in this study were part of the 16.4% shared with humans (28).

Kwon et al. reported that Aa and Pg lacked reliable association with periodontal disease in dogs, as all nine bacterial species (except Aa and Pg) showed a significant correlation with periodontitis in comparisons between the reversible and irreversible groups and each PD group (11). In this study, which used a much larger sample size than previous studies, Pg showed statistical significance for periodontal disease in dogs, unlike Aa. The odds ratio for the other 10 bacteria indicated that an increase in the number of bacteria was associated with the appearance and severity of periodontal disease; however, Pg had an odds ratio of less than 1, suggesting an association between decreased Pg count and periodontal disease. The overall prevalence of Pg was low at 9.87%, with only a 1.12-fold difference in prevalence between the reversible and irreversible groups. This finding significantly differed from the results of Ozavci et al., who reported an 88% prevalence of Pg in 51 dogs with periodontal disease (33). This difference is likely due to the significantly varying sample sizes.

The bacterial prevalence patterns observed in the present study were remarkably similar to the previous study, which examined the overall prevalence of 11 bacteria, excluding *P. gulae* (11). The overall prevalence of Ec was the highest in both studies, at 90.42 and 86.90%, respectively. Moreover, the overall prevalence of *P. gulae* (newly included in this study) ranked second-highest at 76.85%. A comparison of the differences in prevalence between the reversible and irreversible groups revealed a similar pattern, except for Aa and Pn. Pn exhibited an almost 10-fold difference in prevalence in this study compared to the previous study. *P. gulae* was highly prevalent in both reversible and irreversible groups. Also, the difference in prevalence was only 1.30 times.

The overall bacterial count in both studies exhibited a similar pattern, with Aa having the lowest bacterial count in both cases, while *P. gulae* displayed the highest count (9,144,012) in this study. Comparing the difference in bacterial counts between the reversible and irreversible groups, Td showed the highest numerical difference in both studies, with a 146.29-fold increase in this study, significantly higher than the previous study (24.58-fold). Conversely, *P. gulae* had a 3.13-fold difference in bacterial abundance alongside Aa, Pg, Tf, Fn, and Ec in this study. Both studies also examined the differences in bacterial counts between the reversible group and each PD group; Td showed the highest difference in bacterial counts compared to each PD group in both studies. Specifically, in this study, Td exhibited differences of over 100-fold in each PD group.

In contrast to the previous study on the combinations of nine bacterial species, this study examined the association of the abundance of 11 combined pathogens (excluding Aa) between the reversible and irreversible groups, each PD group, and between PD groups. Previous research suggested that Td and Pi could be prognostic biomarkers for periodontitis in dogs (11). In addition to Td and Pi, the results of the present study underscored that Pg, Tf, Td, Fn, and Pi were statistically significant for association and correlation across all comparison conditions. These results strongly suggested that Tf, Td, Fn, and Pi had a robust association and correlation with the severity of periodontal disease in dogs, while Pg showed relatively weak significance.

Furthermore, this study examined the association between PD groups. Fn demonstrated strong significance between PD0 and PD1, PD1 and PD2, and PD2 and PD3, implying that Fn could be a meaningful biomarker for the severity of periodontal disease in dogs. Td showed a very strong association between PD1 and PD2, suggesting that Td could be a useful biomarker between reversible and irreversible clinical stages. However, none of these bacteria showed a statistical association between PD3 and PD4, probably because the disease had progressed significantly over a long period, leading to environmental changes, which may reduce the discriminatory power of the markers.

Several studies have reported the prevalence of *P. gulae* (rather than Pg) is periodontal disease in dogs. Previous research has suggested that *P. gulae* is uncommon in humans but frequently found in animals with periodontitis (29, 34). Some studies have even proposed a probable association between *P. gulae* and periodontal disease in dogs (12, 35). Our findings revealed a notably high overall prevalence of *P. gulae* in dogs, reaching 76.85%. This was similar to the findings of previous studies (36, 37). However, when comparing the reversible and irreversible groups, the prevalence difference was minimal, with only a 1.30-fold change. While statistical significance was observed for individual bacterial counts and their association with the severity of periodontal disease, the correlation and association of bacterial counts in the combination of 11 bacteria (excluding Aa) between the reversible and irreversible groups and each PD group did not exhibit statistical significance. Moreover, comparisons between each periodontal disease group did not yield significant results. Based on these findings, the present study suggests that *P. gulae* may not play an important role in the severity of periodontal disease in dogs unlike previous studies (12, 29, 31, 34–37).

Fn is a Gram-negative, anaerobic bacterium identified in subgingival plaque from dogs with and without periodontitis (30). The pathogenicity mechanisms of Fn are still unclear (32). However, the role of Fn with periodontal disease could be temporary between Gram-positive and Gram-negative bacteria, similar to a bridge between early bacterial colonizers and late bacterial colonizers in the human subgingival space (38, 39). Td is also a Gram-negative, anaerobic bacterium, with significantly higher counts in dogs with periodontitis than those without (11, 32). Td produces virulence factors, such as mobility and chemotactic factors, which allow the bacterium to rapidly colonize new sites, penetrate deep periodontal pockets, and penetrate epithelial layers (40, 41). Cell surface proteins cause dysregulation of host defense, thereby protecting the subgingival biofilm and causing host tissue destruction (30, 31). The role of these two bacteria in canine periodontal disease has not yet been determined. However, this study has shown that they could be useful in predicting the severity of periodontal disease, and further research on this topic is needed in the future.

This study suggests that Fn and Td could be robust biomarkers for periodontal disease in dogs under 20 kg. Fn is a more appropriate biomarker for gingivitis and periodontal disease, whereas Td is a more suitable biomarker for periodontitis (irreversible periodontal disease) in small dogs. A limitation of this study is that it focused on a limited set of target teeth, restricted the sample to medium-sized and smaller dogs, and did not analyze the correlation with breeds.

## 5 Conclusion

Of the 12 putative periodontopathic bacteria analyzed in this study, Aa was not significantly associated with periodontal disease in dogs, while the remaining 10 bacteria showed a significant association. Interestingly, *P. gulae* did not play a crucial role in the severity of periodontal disease; however, Fn and Td were important contributors to the periodontal disease. Therefore, Fn and Td could serve as robust biomarkers for the severity of periodontal disease in small dogs.

## Data Availability

The original contributions presented in the study are included in the article/supplementary material, further inquiries can be directed to the corresponding author/s.

## Footnotes

^1^MedCalc Software Ltd., Ostend, Belgium; https://www.medcalc.org; 2023.
